# Surgical Resection of a Refractory Lung Abscess Secondary to Classic Hodgkin Lymphoma

**DOI:** 10.1016/j.atssr.2025.04.026

**Published:** 2025-05-27

**Authors:** Wakako Nagase, Junichi Maeda, Shigeki Morita, Shunsuke Shigefuku, Tatsuhiro Hoshino, Yuji Minegishi, Shingo Ikeda

**Affiliations:** 1 Department of Thoracic Surgery, Mitsui Memorial Hospital, Tokyo, Japan; 2 Department of Pathology, Mitsui Memorial Hospital, Tokyo, Japan; 3 Department of Respiratory Medicine, Mitsui Memorial Hospital, Tokyo, Japan

## Abstract

Lung abscesses pose diagnostic and management challenges, particularly when they are associated with underlying causes, such as lymphoma. We report the case of a previously healthy adolescent with a lung abscess and lymphadenopathy. Despite antibiotics, improvement was minimal, and biopsy results were inconclusive. Surgery revealed an enlarged, adherent lymph node. Pathologic examination confirmed classic Hodgkin lymphoma invading the hilum and causing bronchial and vascular obstruction and secondary abscesses. This case highlights the importance of including lymphoma in the differential diagnosis of persistent lung infections and the surgical challenges arising from lymphoma-related lymph nodes with irregular enlargement and firm adhesion to surrounding tissues.

Classic Hodgkin lymphoma (CHL) is most common in young and middle-aged adults and often manifests with asymptomatic mediastinal lymphadenopathy.^1^ Nodular sclerosis CHL (NS-CHL) is the most common subtype, with mediastinal involvement in approximately 60% of cases.^2^ Lymphadenopathy in the mediastinum can cause secondary complications such as obstructive pneumonia or lung abscess.

We report a case of NS-CHL diagnosed after surgical resection of a lung abscess. This case presents significant diagnostic and surgical challenges because of hilar obstruction secondary to lymphadenopathy. Written informed consent was obtained from the patient.

A 27-year-old woman presented with worsening productive cough and fever. Two months before presentation, she first noticed the symptoms and was initially treated for presumed pneumonia, but antibiotics failed to alleviate her symptoms. Chest radiography showed worsening of the infiltrative shadows, thus prompting referral for further evaluation and treatment. Her medical history included childhood pneumonia and smoking tobacco (one-half pack/d for 7 years). Physical examination revealed a body temperature of 37.1 °C, decreased breath sounds in the right upper lung field, and painless swelling of the right supraclavicular lymph nodes. She denied night sweats or any recent weight loss.

Laboratory findings included leukocytosis (white blood cells, 20,000/μL; neutrophils, 18,060/μL), anemia (hemoglobin, 11.7 g/dL), thrombocytopenia (41,500/μL), and elevated inflammatory marker levels (C-reactive protein, 8.72 mg/dL; interleukin-2 receptor, 908 U/mL). Results of tumor marker tests, HIV antigen and antibody tests, and T-cell spot of tuberculosis assay were negative, as were bacteriologic and mycologic cultures.

Chest radiography revealed consolidation of the right upper lung field (Figure 1A). Computed tomography revealed a 6 cm × 4 cm cavitary lesion with an air-fluid level in the right upper lobe, along with mediastinal, hilar, and right supraclavicular lymphadenopathy. The bronchus of the right upper lobe showed compressive stenosis caused by enlarged lymph nodes (Figures 1B, 1C).

**Figure 1:**
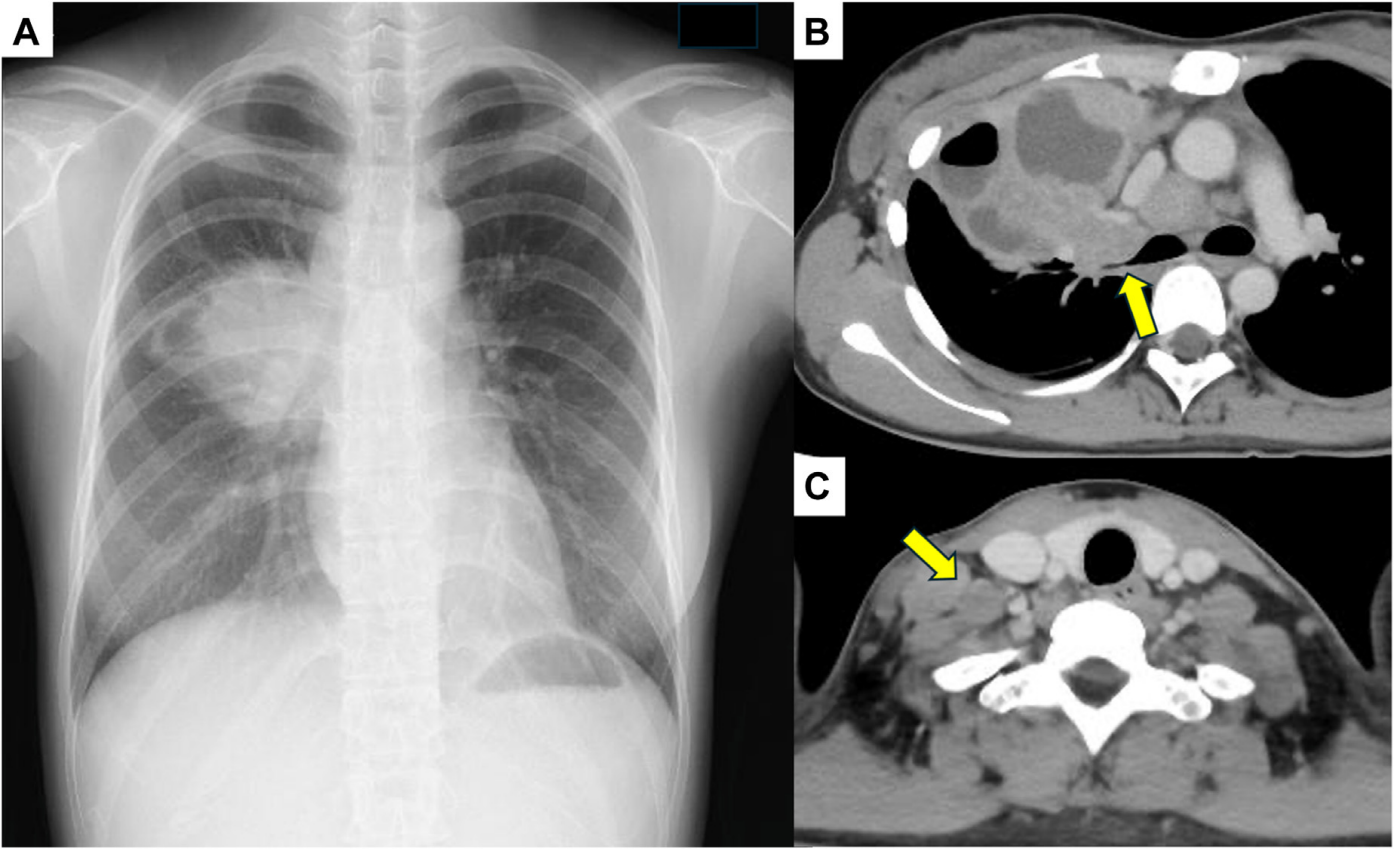
Initial imaging findings. (A) Chest radiography revealed consolidation in the right upper lung field. (B) Chest computed tomography demonstrated a cavitary lesion with an air-fluid level in the right upper lobe. The right upper lobe bronchus showed compressive stenosis caused by lymphadenopathy (yellow arrow). (C) Right supraclavicular lymphadenopathy was observed (yellow arrow).

Bronchoscopy revealed narrowing of the right B3 bronchus with mucosal edema. Transbronchial lung biopsy from the B3 bronchus and endobronchial ultrasound-guided fine-needle aspiration of the paratracheal lymph nodes (#4R) were performed but showed no evidence of malignancy. On the basis of the clinical and radiologic findings, a lung abscess was suspected, and empiric antibiotic therapy was initiated. Despite antibiotic therapy, the patient’s fever and hyperleukocytosis persisted, and surgical resection was performed to investigate the possibility of malignancy and manage the lung abscess.

The 4-port video-assisted thoracoscopic surgical procedure revealed a necrotic mass with dense inflammatory adhesions, extensive fibrosis, and enlarged lymph nodes (#10-12) (Figure 2). Given these findings, anatomic identification was difficult, and the surgical procedure was challenging. Analysis of frozen sections of the lymph nodes revealed no malignancy. We identified the vessels and separated the nodules. Right upper lobectomy with partial resection of the middle lobe was performed. Complete lymph node resection was not feasible because of extensive fibrosis.

**Figure 2:**
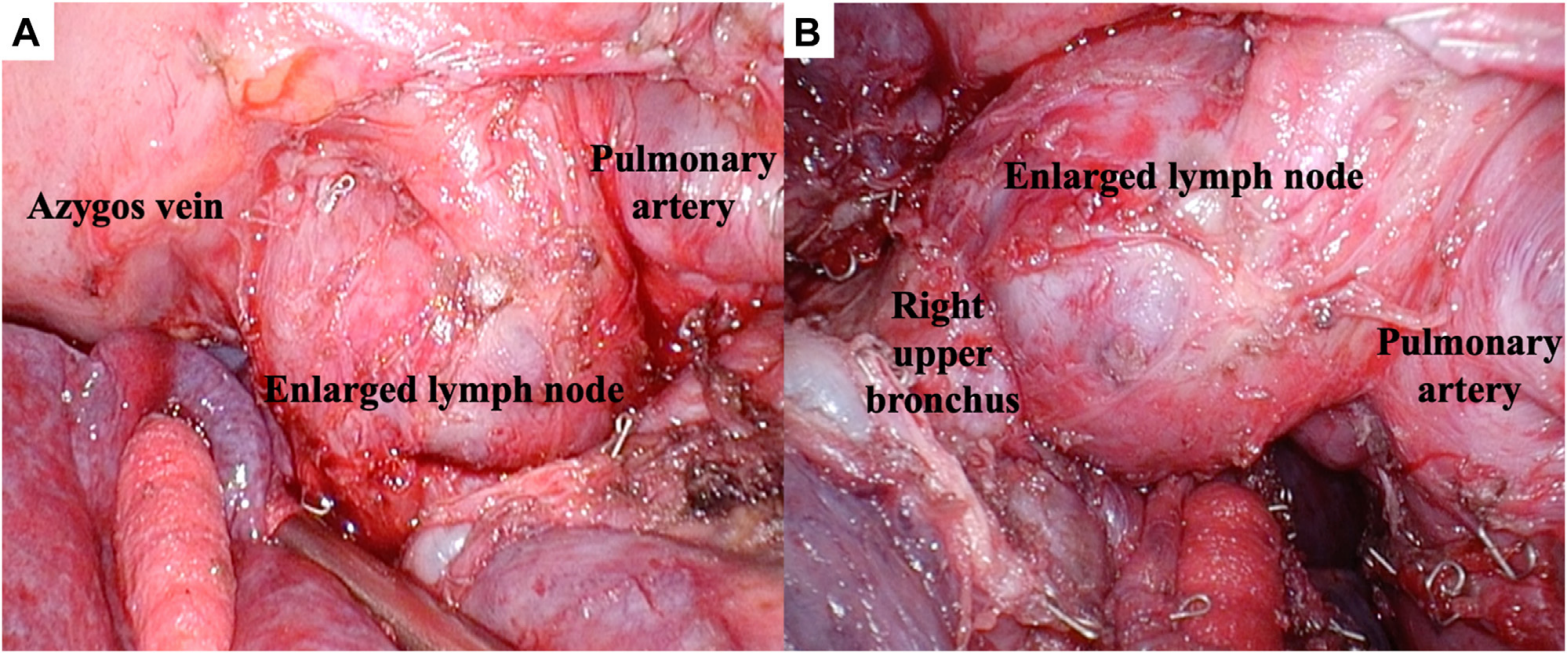
Intraoperative findings. (A) The hilar lymph nodes were markedly enlarged, with severe fibrosis and sclerosis resulting in firm adherence to surrounding structures. (B) This enlarged lymph node with fibrosis caused compression to the adjacent artery and bronchi.

The excised right upper lobe measured 15 cm × 10 cm × 4.5 cm and exhibited notable pathologic features, including areas of collapse, necrosis, cavitation, and hemorrhage, primarily within segment 3 (Figure 3A). The hilum was surrounded by dense sclerosis, which caused obstruction of the peripheral vessels and bronchi, leading to obstructive pneumonia.

**Figure 3:**
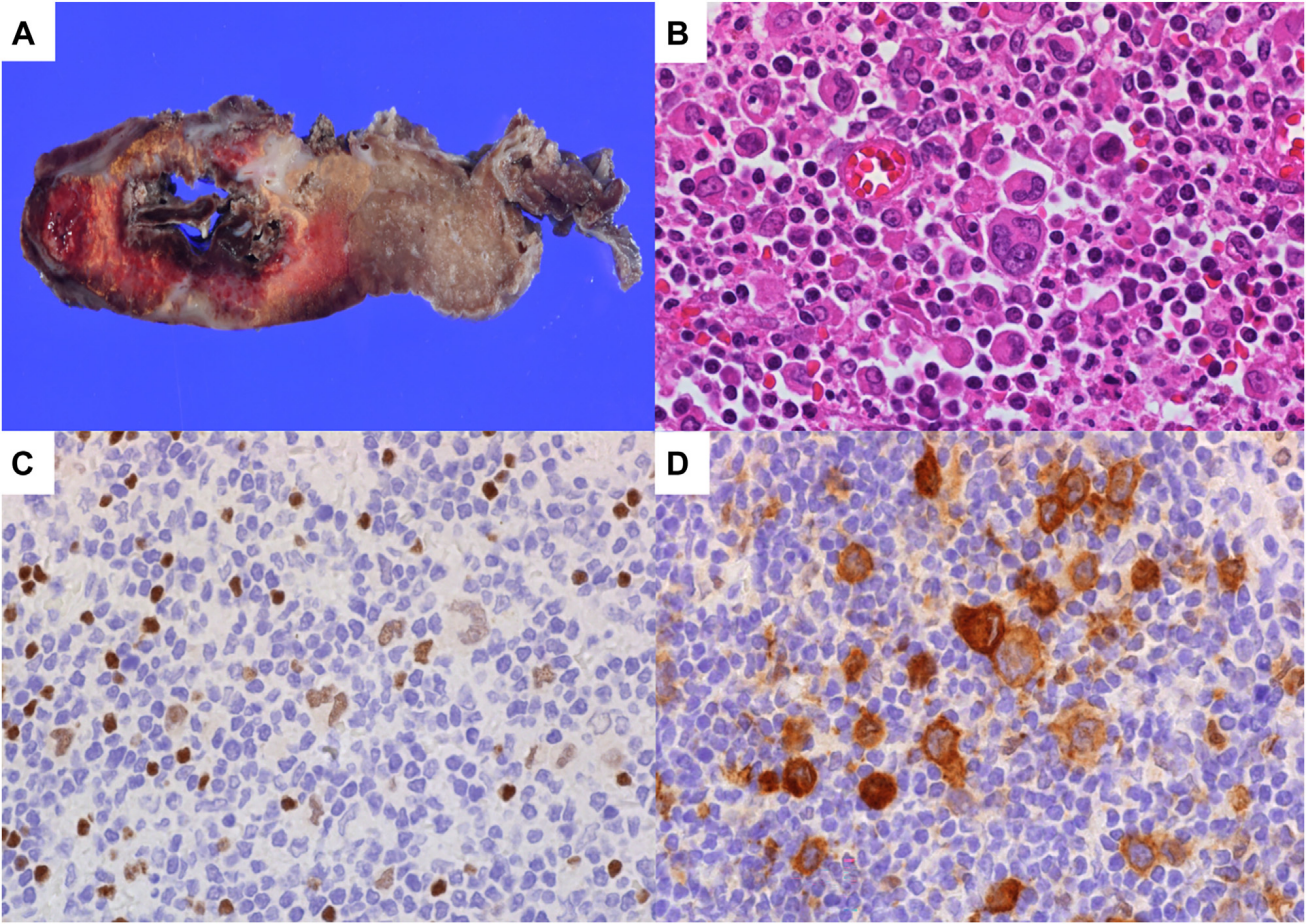
Macroscopic and microscopic tumor features. (A) The excised right upper lobe showed areas of necrosis and bleeding, mainly in segment 3. (B) Microscopic analysis revealed large mononuclear and multinuclear cells, including Hodgkin Reed-Sternberg (HRS) cells with prominent nucleoli. (Hematoxylin and eosin; original magnification ×400.) (C) HRS cells were weakly positive for PAX5. (PAX5; Original magnification ×400.) (D) HRS cells were positive for CD30 (CD30; Original magnification ×400.)

Histopathologic examination showed extensive inflammatory cell infiltration, thick collagenous bands, and Hodgkin Reed-Sternberg cells. Lacunar cells, identified by voids surrounding their nucleoli, were also present (Figure 3B). Immunohistochemical analysis revealed that the Hodgkin Reed-Sternberg cells were positive for PAX5 and CD30 and negative for CD3 and CD20. The result of in situ hybridization for Epstein-Barr virus–encoded RNA was negative (Figures 3C, 3D). These findings were diagnostic of NS-CHL. The final diagnosis suggested CHL of mediastinal origin that had extended to the hilum of the right upper lobe, thereby causing a secondary abscess and cavitation.

After surgical resection, the patient’s symptoms improved significantly, and the patient was discharged 13 days postoperatively. The patient is currently undergoing treatment for NS-CHL.

## Comment

This case underscores the importance of considering lymphomas, particularly NS-CHL, in the differential diagnosis of lung abscesses. Tumors tend to grow with invasion and sclerosis, and these features highlight the potential challenges associated with surgical management.

Lung abscesses are localized areas of necrosis with lung tissue inflammation. The appearance of a pulmonary cavity with air-fluid levels on radiographic imaging is frequently associated with lung abscesses, which are primarily caused by central necrosis of the lung tissue. Although rare, lung abscesses can occur as a secondary complication of lymph node involvement in lymphomas. Lung abscess formation is typically observed in patients with a diagnosis of CHL who are undergoing treatment for advanced or recurrent stages with significant mediastinal lymphadenopathy.^2^^,^^3^ This case of CHL is unusual because of its initial manifestation as a lung abscess at the patient’s first visit, which led to significant diagnostic challenges. CHL was confirmed only after surgical resection because preoperative biopsy did not provide a diagnosis of CHL. This finding is consistent with studies noting the difficulty of diagnosing malignant lymphoma by using small tissue samples, which often lack sufficient malignant cells for a definitive diagnosis.^4^ Furthermore, overlapping infectious symptoms mask the typical signs of lymphoma, such as fever, sweating, and fatigue, thus further complicating diagnosis.

Standard treatment for lung abscesses is management with systemic antibiotics. Surgical interventions are recommended for refractory or severe cases with complications or suspected malignancy.^5^ However, surgery can be unexpectedly difficult given underlying causes such as lymphoma. NS-CHL often exhibits invasive growth patterns, extending into surrounding tissues, such as the chest wall and blood vessels.^6^ In the present case, the nodules extended into the hilum and compressed the bronchi and adjacent vascular structures, thereby complicating the identification of anatomic structures. Understanding the specific characteristics of NS-CHL is essential because unlike in other lymphomas or inflammatory lymphadenopathy, lymph node resection from surrounding structures can be challenging, making surgical planning for pneumonectomy or conversion to thoracotomy crucial. A thorough assessment of potential causes, including CHL, alongside an understanding of tumor-specific characteristics, is essential for surgical planning.

In conclusion, this case highlights the diagnostic challenges associated with refractory lung abscesses, particularly when underlying malignant diseases such as NS-CHL are involved. Diagnosis can be particularly challenging because of the overlapping clinical features of lymphoma and lung abscess. This case underscores the importance of including lymphoma in the differential diagnosis of persistent lung infections in young adolescents with enlarged lymph nodes who are unresponsive to standard treatments. Moreover, surgical intervention is expected to be more challenging than typical lung abscess procedures because of the invasive growth pattern of NS-CHL and requires careful preoperative planning.

## References

[1] Ansell S.M. (2015). Hodgkin lymphoma: diagnosis and treatment. Mayo Clin Proc.

[2] Bae Y.A., Lee K.S. (2008). Cross-sectional evaluation of thoracic lymphoma. Radiol Clin North Am.

[3] Brennan D.D., Gleeson T., Coate L.E., Cronin C., Carney D., Eustace S.J. (2005). A comparison of whole-body MRI and CT for the staging of lymphoma. AJR Am J Roentgenol.

[4] Farmer P.L., Bailey D.J., Burns B.F., Day A., LeBrun D.P. (2007). The reliability of lymphoma diagnosis in small tissue samples is heavily influenced by lymphoma subtype. Am J Clin Pathol.

[5] Kuhajda I., Zarogoulidis K., Tsirgogianni K. (2015). Lung abscess-etiology, diagnostic and treatment options. Ann Transl Med.

[6] Witte B., Hürtgen M. (2006). Lymphomas presenting as chest wall tumors. Thorac Surg Sci.

